# Occurrence of selected Covid-19 drugs in surface water resources: a review of their sources, pathways, receptors, fate, ecotoxicity, and possible interactions with heavy metals in aquatic ecosystems

**DOI:** 10.1007/s10653-024-02293-9

**Published:** 2024-11-28

**Authors:** S. R. Maremane, G. N. Belle, P. J. Oberholster, E. O. Omotola

**Affiliations:** 1 Faculty of Natural and Agricultural Sciences, Centre for Environmental Management, University of the Free State, PO Box 339, Bloemfontein, 9300 South Africa; 2 Department of Chemical Sciences, College of Science and Information Technology, Tai Solarin, University of Education, Ijebu-Ode, Lagos, Ogun State Nigeria; 3 Centre for Mineral Biogeochemistry, University of the Free State, Bloemfontein, South Africa

**Keywords:** Covid-19 drugs, Surface water resources, Emerging pollutants, Combined pollutants, Wastewater effluent, Heavy metals

## Abstract

The outbreak of the coronavirus disease 2019 (Covid-19) led to the high consumption of antibiotics such as azithromycin as well as corticosteroids such as prednisone, prednisolone, and dexamethasone used to treat the disease. Seemingly, the concentrations of these four Covid-19 drugs increased in wastewater effluents and surface water resources. This is due to the failure of traditional wastewater treatment facilities (WWTFs) to eliminate pharmaceuticals from wastewater. Therefore, the objective of the current research was to review the present state of literature on the occurrence of four Covid-19 drugs in water resources, the associated risks and toxicity, their fate, as well as the emergence of combined pollutants of Covid-19 drugs and heavy metals. From late 2019 to date, azithromycin was observed at concentrations of 935 ng/L, prednisone at 433 ng/L, prednisolone at 0.66 ng/L, and dexamethasone at 360 ng/L, respectively, in surface water resources. These concentrations had increased substantially in water resources and were all attributed to pollution by wastewater effluents and the rise in Covid-?19 infections. This phenomenon was also exacerbated by the observation of the pseudo-persistence of Covid-19 drugs, long half-life periods, as well as the excretion of Covid-19 drugs from the human body with about 30?90% of the parent drug. Nonetheless, the aquatic and human health toxicity and risks of Covid-19 drugs in water resources are unknown as the concentrations are deemed too low; thus, neglecting the possible long-term effects. Also, the accumulation of Covid-19 drugs in water resources presents the possible development of combined pollutants of Covid-19 drugs and heavy metals that are yet to be investigated. The risks and toxicity of the combined pollutants, including the fate of the Covid-19 drugs in water resources remains a research gap that undoubtably needs to be investigated.

## German Abstract

Der Ausbruch der Coronavirus-Krankheit 2019 (Covid-19) führte zu einem hohen Verbrauch von Antibiotika wie Azithromycin sowie Kortikosteroiden wie Prednison, Prednisolon und Dexamethason, die zur Behandlung der Krankheit eingesetzt wurden. Anscheinend stiegen die Konzentrationen dieser vier Covid-19-Medikamente in den Abwässern und Oberflächenwasserressourcen an. Dies ist darauf zurückzuführen, dass traditionelle Kläranlagen es nicht geschafft haben, Arzneimittel aus dem Abwasser zu eliminieren. Ziel der aktuellen Forschung war es daher, den aktuellen Stand der Literatur über das Vorkommen von vier Covid-19-Medikamenten in Wasserressourcen, die damit verbundenen Risiken und Toxizität, ihr Schicksal sowie das Auftreten von kombinierten Schadstoffen von Covid-19-Medikamenten und Schwermetallen zu überprüfen. Von Ende 2019 bis heute wurde Azithromycin in Konzentrationen von 935 ng/L, Prednison bei 433 ng/L, Prednisolon bei 0,66 ng/L und Dexamethason bei 360 ng/L in Oberflächenwasserressourcen beobachtet. Diese Konzentrationen waren in den Wasserressourcen erheblich gestiegen und wurden alle auf die Verschmutzung durch Abwässer und den Anstieg der Covid-19-Infektionen zurückgeführt. Dieses Phänomen wurde auch durch die Beobachtung der Pseudopersistenz von Covid-19- Medikamenten, langer Halbwertszeiten sowie der Ausscheidung von Covid-19-Medikamenten aus dem menschlichen Körper mit etwa 30 % bis 90 % des Mutterarzneimittels verschärft. Die Toxizität und die Risiken von Covid-19-Medikamenten in den Wasserressourcen für die aquatische und menschliche Gesundheit sind jedoch nicht bekannt, da die Konzentrationen als zu niedrig eingestuft werden. So werden die möglichen Langzeitwirkungen vernachlässigt. Auch die Akkumulation von Covid-19-Medikamenten in den Wasserressourcen stellt die mögliche Entwicklung von kombinierten Schadstoffen aus Covid-19-Medikamenten und Schwermetallen dar, die noch untersucht werden müssen. Die Risiken und die Toxizität der kombinierten Schadstoffe, einschließlich des Verbleibs der Covid-19-Medikamente in den Wasserressourcen, bleiben eine Forschungslücke, die zweifellos untersucht werden muss.

## French Abtract

L?épidémie de la maladie à coronavirus 2019 (Covid-19) a entraîné une forte consommation d?antibiotiques tels que l?azithromycine ainsi que de corticostéroïdes tels que la prednisone, la prednisolone et la dexaméthasone utilisés pour traiter la maladie. Apparemment, les concentrations de ces quatre médicaments Covid-19 ont augmenté dans les effluents d?eaux usées et les ressources en eau de surface. Cela est dû à l?incapacité des installations traditionnelles de traitement des eaux usées (WWTF) à éliminer les produits pharmaceutiques des eaux usées. Par conséquent, l?objectif de la présente recherche était de passer en revue l?état actuel de la littérature sur l?apparition de quatre médicaments Covid-19 dans les ressources en eau, les risques et la toxicité associés, leur devenir, ainsi que l?émergence de polluants combinés des médicaments Covid-19 et des métaux lourds. De la fin de 2019 à ce jour, l?azithromycine a été observée à des concentrations de 935 ng/L, de 433 ng/L de prednisone, de 0,66 ng/L de prednisolone et de 360 ng/L, respectivement, dans les ressources en eau de surface. Ces concentrations avaient considérablement augmenté dans les ressources en eau et étaient toutes attribuées à la pollution par les effluents d?eaux usées et à l?augmentation des infections au Covid-19. Ce phénomène a également été exacerbé par l?observation de la pseudo-persistance des médicaments Covid-19, des longues périodes de demi-vie, ainsi que de l?excrétion des médicaments Covid-19 du corps humain avec environ 30 à 90 % du médicament parent. Néanmoins, la toxicité et les risques pour la santé aquatique et humaine des médicaments Covid-19 dans les ressources en eau sont inconnus car les concentrations sont jugées trop faibles ; négligeant ainsi les effetspossibles à long terme. Aussi, l?accumulation de médicaments Covid-19 dans les ressources en eau présente le développement possible de polluants combinés de médicaments Covid-19 et de métaux lourds qui doivent encore être étudiés. Les risques et la toxicité des polluants combinés, y compris le devenir des médicaments Covid-19 dans les ressources en eau, restent une lacune de la recherche qui doit sans aucun doute être étudiée.

## Spanish Abstract

El brote de la enfermedad por coronavirus 2019 (Covid-19) provocó un alto consumo de antibióticos como la azitromicina, así como de corticosteroides como la prednisona, la prednisolona y la dexametasona utilizados para tratar la enfermedad. Al parecer, las concentraciones de estos cuatro medicamentos contra el Covid-19 aumentaron en los efluentes de aguas residuales y en los recursos hídricos superficiales. Esto se debe al fracaso de las instalaciones tradicionales de tratamiento de aguas residuales (WWTF) para eliminar los productos farmacéuticos de las aguas residuales. Por lo tanto, el objetivo de la presente investigación fue revisar el estado actual de la literatura sobre la ocurrencia de cuatro medicamentos Covid-19 en los recursos hídricos, los riesgos y toxicidad asociados, su destino, así como la aparición de contaminantes combinados de medicamentos Covid-19 y metales pesados. Desde finales de 2019 hasta la fecha, se observó azitromicina en concentraciones de 935 ng/L, prednisona en 433 ng/L, prednisolona en 0,66 ng/L y dexametasona en 360 ng/L, respectivamente, en recursos de aguas superficiales. Estas concentraciones habían aumentado sustancialmente en los recursos hídricos y se atribuyeron a la contaminación por efluentes de aguas residuales y al aumento de las infecciones por Covid-19. Este fenómeno también se vio exacerbado por la observación de la pseudopersistencia de los medicamentos Covid-19, los largos períodos de vida media, así como la excreción de medicamentos Covid-19 del cuerpo humano con aproximadamente el 30%-90% del medicamento original. No obstante, se desconoce la toxicidad y los riesgos para la salud acuática y humana de los medicamentos contra la COVID-19 en los recursos hídricos, ya que las concentraciones se consideran demasiado bajas; por lo tanto, descuidando los posibles efectos a largo plazo. Además, la acumulación de medicamentos Covid-19 en los recursos hídricos presenta el posible desarrollo de contaminantes combinados de medicamentos Covid-19 y metales pesados que aún no se han investigado. Los riesgos y la toxicidad de los contaminantes combinados, incluido el destino de los medicamentos contra la COVID-19 en los recursos hídricos, siguen siendo una laguna de investigación que, sin duda, debe ser investigada.

## Italian Abstract

L'epidemia di coronavirus 2019 (Covid-19) ha portato all'elevato consumo di antibiotici come l'azitromicina e di corticosteroidi come il prednisone, il prednisolone e il desametasone usati per trattare la malattia. Apparentemente, le concentrazioni di questi quattro farmaci Covid-19 sono aumentate negli effluenti delle acque reflue e nelle risorse idriche superficiali. Ciò è dovuto al fallimento degli impianti tradizionali di trattamento delle acque reflue (WWTF) nell'eliminare i prodotti farmaceutici dalle acque reflue. Pertanto, l'obiettivo della presente ricerca è stato quello di esaminare lo stato attuale della letteratura sulla presenza di quattro farmaci Covid-19 nelle risorse idriche, i rischi e la tossicità associati, il loro destino, nonché l'emergere di inquinanti combinati di farmaci Covid-19 e metalli pesanti. Dalla fine del 2019 ad oggi, l'azitromicina è stata osservata a concentrazioni di 935 ng/L, il prednisone a 433 ng/L, il prednisolone a 0,66 ng/L e il desametasone a 360 ng/L, rispettivamente, nelle risorse idriche superficiali. Questeconcentrazioni sono aumentate notevolmente nelle risorse idriche e sono state tutte attribuite all'inquinamento da acque reflue e all'aumento delle infezioni da Covid-19. Questo fenomeno è stato esacerbato anche dall'osservazione della pseudo-persistenza dei farmaci Covid-19, dei lunghi periodi di emivita, nonché dell'escrezione di farmaci Covid-19 dal corpo umano con circa il 30%-90% del farmaco genitore. Tuttavia, la tossicità e i rischi per la salute acquatica e umana dei farmaci Covid-19 nelle risorse idriche sono sconosciuti in quanto le concentrazioni sono ritenute troppo basse; trascurando così i possibili effetti a lungo termine. Inoltre, l'accumulo di farmaci Covid-19 nelle risorse idriche presenta il possibile sviluppo di inquinanti combinati di farmaci Covid-19 e metalli pesanti che devono ancora essere studiati. I rischi e la tossicità degli inquinanti combinati, compreso il destino dei farmaci Covid-19 nelle risorse idriche, rimangono una lacuna di ricerca che senza dubbio deve essere indagata.

## Introduction

From a global view, Covid-19 infections have subsided, but the effects of Covid-19 therapeutic drugs on the environment and water resources will remain for decades to come. This is driven by the constant use of Covid-19 drugs by patients, and the disposal of wastewater effluents that contain Covid-19 drugs into the environment and water resources. Also, variants of Covid-19 continue to emerge (Bardhan et?al., 2023), thus threatening the possibility of a new Covid-19 wave of infections that will require more Covid-19 medication to treat Covid-19 symptoms. The majority of patients infected by Covid-19 have mild symptoms and mostly recover at home using home-based remedies. Be that as it may, some symptoms are treated with over-the-counter medication, whereas some medications are administered to patients upon hospitalisation (Betancourt et?al., 2021). Several clinical drugs, including azithromycin, prednisone, prednisolone, and dexamethasone, are among those used to treat patients infected with Covid-19 because of their use to treat respiratory diseases such as asthma and lung infections which are similar to Covid-19 (D?Aoust et?al., 2021; Morales-Paredes et?al., 2022). Azithromycin is an antibiotic used to treat Covid-19 because of its ability to act against bacteria that cause respiratory diseases (Morales-Paredes et?al., 2022). More so, azithromycin also has immunomodulatory and antiviral activities that substantially reduce the effects of Covid-19 (Medema et?al., 2020). On the other hand, prednisone is characterised as a corticosteroid due to its ability to reduce the immune system?s response to numerous diseases (Morales-Paredes et?al., 2022). This minimises symptoms such as allergic reactions and swelling which are associated with Covid-19 (Yazdan et?al., 2021). Similarly, prednisolone is also a corticosteroid characterised by its reduction of inflammation in the body, treatment of asthma, and other respiratory diseases such as Covid-19 and autoimmune diseases (Yazdan et?al., 2021). Lastly, dexamethasone, also a corticosteroid, is used for critical Covid-19 patients, particularly children, who need supplemental oxygen (D?Aoust et?al., 2021). This is because of its ability to treat respiratory diseases like asthma and obstructive lung disease (Yazdan et?al., 2021). This is an indication that the treatment of Covid-?19 requires a wide array of pharmaceuticals ranging from antibiotics to corticosteroids that are consumed by patients to manage the symptoms of the disease.

The Covid-19 infections have led to a spike in the use of Covid-19 pharmaceutical drugs, and also some therapeutic drugs were repurposed to treat the disease (Kock et al., 2023). Pharmaceuticals such as prednisone, prednisolone, dexamethasone as well as azithromycin were therefore grouped as Covid-19 drugs due to their ability to treat the Covid-19 disease. This however, does not limit their uses and further resulted in the exposure of such drugs to the environment and water resources in higher concentrations (Hayden et al., 2022). The consumption of Covid-19 drugs increased drastically since the outbreak of the Covid-19 pandemic in March 2020 (Kock et?al., 2023; Kuroda et?al., 2021). On the contrary, some pharmaceutical drugs have demonstrated resilience to being metabolised by the human body (Netshithothole & Madikizela, 2024; Hayden et?al., 2022; Niang et?al., 2023). Thus, they are released into domestic wastewater treatment facilities through human excretion in almost their parent form (Hayden et?al., 2022; Netshithothole et?al., 2024; Niang et?al., 2023; Waleng & Nomngongo, 2022). Domestic WWTFs have proven to fail to remove such organic pollutants, as well as inorganic pollutants such as heavy metals, which have resulted in the release of treated wastewater effluents that contain pharmaceutical drugs and heavy metals into environmental water resources (Kock et?al., 2023; Maremane et al., 2024; Niang et?al., 2023).

Little is known about the extent of pollution of surface water resources by Covid-19 drugs. For this reason, pharmaceutical pollution of water resources by Covid-19 drugs is underrepresented in the scientific body of knowledge. Thus, this study reviewed the sources, pathways, concentrations, distribution, fate, and receptors of Covid-19 drugs in environmental surface water resources. Traces of Covid-19 drugs such as azithromycin (Morales-Paredes et?al., 2022; Pan & Yau, 2021), prednisone (Gong et?al., 2019), and dexamethasone (He et?al., 2022) have been observed in wastewater effluents, but there is limited research work to ascertain the prevalence of these Covid-19 drugs in environmental surface water resources. Moreover, prednisolone, also a Covid-19 drug, has a minute literature background of being assessed in wastewater effluents and water resources, particularly after the Covid-19 outbreak in 2020. To date, research works have concluded that exposures to environmental pharmaceutical residues do not affect human health after determining the risks and ecotoxicity by the use of fish bioassays (Medema et?al., 2020; Morales-Paredes et?al., 2022; Singer et?al., 2023). But the studies overlooked the possible bioaccumulation of the drugs toxins in the human body due to minimal exposure over long periods. The fact that research works have concluded that there are no risks posed to human health does not rule out the possibility of being toxic to aquatic life. Most times, these pollutants bioaccumulate in the bodies of the aquatic organisms. Consequently, when these lower organisms are being fed on by humans, the pollutants bio-magnify in human bodies, and this might lead to chronic adverse effects. It is therefore imperative to expand scientific knowledge concerning the risks and toxicity of pharmaceuticals, particularly Covid-19 drugs on human health. As a result, a novel study that uses fish embryos as bioassays to investigate and demonstrate the direct health implications of pharmaceuticals, in particular Covid-19 drugs on human health, is needed. This is because fish embryos efficiently demonstrate the adverse effects of test compounds on critical developmental processes and are sensitive to very low dosages of toxins (Capela et?al., 2020). Additionally, fish embryos have similar genetic data to most animals and humans (Chahardehi et?al., 2020). Also, fish embryos are produced in large numbers, therefore, they allow for large samples and are then statistically powerful for dose?response studies (Truong et?al., 2011). Despite all the advantages of using fish embryos for chemical toxicity tests, to date, no study has explored fish embryos for toxicity testing of pharmaceuticals. The observations in the changes of the fish embryos after exposure to Covid-19 drugs will be more precise in demonstrating the human health toxicity and risks associated with Covid-19 drugs. On the other hand, numerous studies have demonstrated the ecotoxicity of pharmaceuticals on aquatic life through indicators such as fish and algae (Ghannam, 2021; Hayden et?al., 2022; Kock et?al., 2023; Kuroda et?al., 2021; Waleng & Nomngongo, 2022). The risks and toxicity observed in the aforementioned studies included cancer, impaired reproduction and growth, and also reduced lifespan of the aquatic life. However, fish and algae can be resistant to toxins at low dosages (Ghannam, 2021; Pemberthy et?al., 2020). As such, the results presented in the previous studies were particularly limited. There is a need to ensure that the risks and ecotoxicity of Covid-19 drugs are extensively determined. Therefore, a novel study that utilises yeast bioassays to assess the ecotoxicity of Covid-19 drugs on aquatic life is needed. This is because yeast is highly sensitive to low concentrations of toxins (Balsiger et?al., 2010; Vyatchina et?al., 2019), and can detect unknown toxins (Ghannam, 2021), and is highly specific to toxins of research interest (Balsiger et?al., 2010).

Exposure of Covid-19 drugs to aquatic life and humans can be harmful and ultimately lethal leading to short and long term negative health conditions to human health and the aquatic life. The effects and lethality of the Covid-19 drugs depends on the dosage, exposure route, and contact time (He et al., 2022). Morales-Paredes et?al. (2022) reported concentrations of azithromycin in surface water in Spain, which increased 217 times during the Covid-19 pandemic. Pan and Yau (2021) also observed azithromycin concentrations ranging from 571 to 1?046 ng/L in global WWTFs On the other hand, Yazdan et?al. (2021) observed prednisone concentrations of 0.05?433 ng/L in surface water. Similarly, Gong et?al. (2019) conducted a study in China, Spain, Japan, France, and the Netherlands where concentrations of prednisone of 0.03?29 ng/L were recorded. The highest observed value of prednisone was 230 ng/L. For dexamethasone, He et?al. (2022) reported concentrations ranging from 0.02 to 0.09 ng/L in wastewater effluents in China. To add to this, Musee et?al. (2021) recorded surface water concentrations of dexamethasone of 147 ng/L in Switzerland, 353 ng/L in Portugal, and 360 ng/L in Spain. There is a need for the determination of long-term risks of Covid-19 drugs due to minimal exposure to water resources, aside from the observation of prednisone in wastewater effluents and water resources (Gong et?al., 2019; Yazdan et?al., 2021). Prednisolone has only been reported in wastewater effluent by Inarmal and Moodley (2023). To date, post-Covid-19, there is a need to efficiently demonstrate the occurrence of prednisolone in effluent of WWTFs and surface water. Therefore, the emergence of new pollutants such as Covid-19 drugs in water resources and the environment needs to be addressed as to ascertain the sustainability and ecological activities of the aqueous and terrestrial environment.

The understanding of the fate of Covid-19 drugs in water resources is unknown. For example, Pei et?al. (2022) assessed the fate of antibiotics and personal care products in water resources and sediments. This was done by determining the leaching potential to groundwater for antibiotics and personal care products. Pei et?al. (2022) further determined the understanding of the solid and liquid phases of antibiotics and personal care products. Understanding the solid and liquid phases was attained by determining the pseudo-partitioning coefficient (P-PC) to achieve a quantitative knowledge of pharmaceuticals in the water and sediment phases. No study could be found that provided an understanding of the relationship of solid and aqueous phases of any pharmaceuticals, including Covid-19 drugs. The phases are represented by the sediment and water phases, which need to be investigated. Moreover, the leaching potential of Covid-19 drugs from sediments into the groundwater is unknown. It is essential to indicate the potential of groundwater pollution by Covid-19 drugs as a result of the pollution of the surface water and sediments by Covid-19 drugs.

The continuous spread of both pharmaceuticals and heavy metals in water resources and sediments presents the development of new pollutants. These new pollutants are a mixture of pollutants which form transformed or mixed pollutants. The most recent studies on possible mixed pollutants by Zhai et?al. (2023) and Zhao et?al. (2023) have demonstrated the simultaneous occurrence of high concentrations of both heavy metals and pharmaceuticals in water resources and sediments. Heavy metals in water resources are mostly attributed to industrialization, urbanization as well as climate change (Aziz et?al., 2023; Zhao et?al., 2023). These are the primary sources that cause the spread of a variety of heavy metals in the environment and water resources. This suggested the possibility of mixed pollutants in water resources and sediments. However, these studies have not ascertained the occurrence of such mixed pollutants and the resultant compounds from the combination of heavy metals and pharmaceuticals. As such, these mixed pollutants could exhibit some level of synergistic adverse effects and consequently be more persistent and harmful (Mukherjee et?al., 2022; Zhai et?al., 2023; Zhao et?al., 2023). Therefore, progressive research is needed to identify mixed pollutants and their characteristics, particularly combined contaminants of Covid-19 drugs and heavy metals.

As more concentrations of Covid-19 drugs are introduced into water resources, the reported abundance of nutrients in surface water resources may play a role in the bioavailability of the Covid-19 drugs and heavy metals. By way of illustration, Miranda et?al. (2022) outlined that there is limited knowledge of the role of nutrients like phosphorus and nitrogen on the possible bioavailability of heavy metals in water resources. On the other hand, Qi et?al. (2022) showed that heavy metals in water resources inhibit the uptake of nutrients by aquatic plants. This essentially demonstrates the parallel occurrence of nutrients and heavy metals in water resources. This results in an abundance of nutrients in water resources. But to date, no study has investigated the influence of nutrients on the bioavailability of heavy metals in surface water. Again, the constant release of pharmaceuticals into water resources that are nutrient-rich requires an investigation into the influence of nutrients on the bioavailability of pharmaceuticals such as Covid-19 drugs. Such knowledge could prove significant as the primary mechanism of adsorption of chemical compounds such as Covid-19 drugs and heavy metals may be enhanced by the abundance of nutrients. This will cause minimal flushing of water resources and also an abundance and recirculation of Covid-19 drugs and heavy metals in water resources. This is detrimental to the sustainability of water resources and more so, toxic to aquatic life and human health.

## Methodology

The consulted literature was selected using Scopus and Google Scholar databases. A total of 107 peer-reviewed papers were consulted. The search focused on keywords such as Covid-19 drugs, antibiotics, corticosteroids, pharmaceuticals, heavy metals, water resources, water pollution, azithromycin, prednisone, prednisolone, dexamethasone, wastewater effluent, wastewater treatment facilities, occurrence, fate, distribution, combined pollutants, emerging pollutants, sources, pathways, receptors, aquatic ecosystems, risk, and toxicity. This was done to ensure that all research papers used were relevant to the focus objective of this review paper. To further ensure the relevance of the selected papers for the literature review, the titles, abstracts, and keywords of the papers were critically reviewed. All papers not meeting the selection criteria were excluded. Also, much attention was given to recent papers published between the years 2019 and 2024 presenting the progression and trends of Covid-19 and its therapeutic drugs in wastewater effluents and water resources. This was necessary to ensure that the papers included the relevant information as well as the most recent research interests and gaps related to Covid-19 drugs in water resources. Ultimately, the selected peer-reviewed literature was used to develop this current review paper.

## Sources and pathways of Covid-19 pharmaceuticals in water resources

### Sources and pathways

#### Point and non-point sources of pharmaceutical pollution

Point sources of pollution by pharmaceuticals include wastewater, sludge or biosolids, solid waste disposal sites that include pharmaceuticals, health care facilities like hospitals and quarantine facilities, and pharmaceutical industries (Gwenzi et?al., 2022; Hayden et?al., 2022; Das et al., 2023; Morales-Paredes et?al., 2022). By way of illustration, Braine et?al. (2024) demonstrated in their study that the land application of biosolids and sewage effluent directly introduces high concentrations of pharmaceutical drug pollutants into the soil, and then to water resources. Inversely, Gwenzi et?al. (2022) found that hospital wastewater was the main source of pharmaceutical pollution into domestic WWTFs, and from there into water resources. This results from clinical wastewater that is often conveyed to nearby urban WWTFs. The production process of these pharmaceuticals also leads to the production of wastes and metabolites of these drugs, which ultimately end up in domestic wastewater (Gwenzi et?al., 2022; O?Flynn et?al., 2021). Therefore, pharmaceutical industries exacerbate water resource pollution by pharmaceutical drugs. O?Flynn et?al. (2021) further demonstrated that pharmaceutical drugs are not entirely metabolised by the human body; thus, they are excreted in about 30?90% of their parent product. This finding shows that wastewater contains a large percentage of the pharmaceutical parent drug. Henceforth, this assertion makes it more challenging to remove pharmaceuticals from wastewater using the traditional domestic wastewater treatment process. This further corroborated the need to advance wastewater treatment technologies to enhance wastewater effluent quality. This will demonstrate adaptation to the rapidly evolving pollutants in wastewater.

Aquaculture and agriculture undoubtedly contribute to the pollution of water resources by pharmaceutical drugs and are deemed non-point sources of pollution (Kock et?al., 2023). This is because of the use of antibiotics and other pharmaceuticals in aquaculture and agriculture that are necessary for the management of diseases and infections in animals and plants (Chandra et?al., 2023). To illustrate this, the Honghu Lake in China, which is a high-intensity aquaculture lake, was analysed for antibiotics, and 13 of the antibiotics were detected with concentrations recorded just above 2?796,6 ng/L (Wang et?al., 2017). Although this was regarded as a low concentration, there is a knowledge gap in terms of long-term exposure to low concentrations of pharmaceuticals in aquatic organisms and humans. Therefore, these studies showed that aquaculture and agriculture play a role in the pharmaceutical pollution of water resources. This pollution may compound over time, making it unmanageable and threatening to the holistic environment and its constituents. The management of antibiotic pollution of the environment from agricultural activities is vital to minimise the possibility of animals becoming resistant to antibiotics.

The indirect pollution of water resources by non-point sources of pollution of pharmaceuticals is not negligible as it plays a role in the accumulation of a variety of pharmaceuticals in water resources. Surface runoff from contaminated soils that were irrigated by sewage effluent, and eroded soils that were conditioned by biosolids that were contaminated by pharmaceuticals, play a key role as the main non-point sources of pollution (Sabourine et?al., 2009). Mirzaie et?al. (2022) found that the shores of Bushehr port in the Bushehr coastline of Persia had high concentrations of azithromycin. This was because the shores received runoff from the city that contained treated sewage. Also, an agricultural site close to the shore used treated sewage for irrigation, after which the eroding soil and sediments, together with the runoff, polluted the sea with pharmaceuticals. Similarly, Sabourine et?al. (2009) observed pharmaceutically contaminated runoff due to the application of municipal biosolids for soil conditioning. These studies then suggested that surface runoff is a non-point source of pharmaceutical pollution to water resources. However, the literature showed a lack of research in recent years on runoff analysis as a possible source of pharmaceutical contamination of water resources. This could be caused by the seasonal occurrence of runoff. Also, these findings substantiate the need to test and pre-treat biosolids before application as soil conditioners due to their contribution as non-point pharmaceutical contamination sources.

#### Pathways of Covid-19 pharmaceutical drugs into water resources

The pathways of pharmaceutical drugs into the aquatic environment are the determining factor of their concentrations (Robson et?al., 2020). Because of this assertion, pathways of pharmaceuticals into water resources have received attention in the previous years, particularly after the Covid-19 pandemic caused an increase in the consumption of pharmaceutical drugs. This is because pathways are the regulators of how the pollutant travels, transforms, and ultimately, its concentration when the pollutant reaches the receptor (Gwenzi et?al., 2022). Worldwide, various forms of wastewater have been presented as the main source of pharmaceutical contamination (Hayden et?al., 2022; Morales-Paredes et?al., 2022; Niang et?al., 2023). Consequently, the resulting large quantities of wastewater effluents generated daily after the treatment of wastewater become the main pathway of pharmaceutical pollution in water resources. This is because the majority of the WWTFs dispose of their treated effluent directly into surface water resources (Gwenzi et?al., 2022; Maremane et?al., 2024), where else they cannot efficiently treat pharmaceuticals from wastewater (Niang et?al., 2023). For this reason, wastewater effluents are observed as the main pathway of pharmaceutical pollution of water resources.

The continuous discharge of wastewater effluents into water resources has presented a challenge in terms of pollution of water resources by pharmaceuticals. For example, a study on wastewater effluents of two facilities in central Pennsylvania revealed that the WWTFs produced effluents having removed just less than 30% of the initial concentration of the pharmaceutical that was received from the influent (Hayden et?al., 2022). Likewise, Kuroda et?al. (2021) assessed wastewater effluent that was discharged into environmental water. The results suggested that the WWTFs only removed less than 20% of the drug concentrations received. This resulted in high pharmaceutical residues in the wastewater effluent. Again, Tarazona et?al. (2021) demonstrated that wastewater effluent from WWTFs generally contains 67% of the pharmaceutical drugs excreted by patients. Niang et?al. (2023) assessed the occurrence of azithromycin from wastewater effluent and the concentrations were considered non-negligible. These studies suggested that pharmaceuticals, including Covid-19 drugs, have a novel persistence to conventional wastewater treatment, and therefore expose water resources to pharmaceutical drugs through the wastewater effluent.

Other potential pathways of pharmaceutical pollution include direct infiltration of contaminated water, accidental spillages of pharmaceuticals, dumpsite leachates, and surface water and groundwater interactions (Kuroda et?al., 2021; Niang et?al., 2023). Moreso, pathways such as dumpsite leachates and accidental spillages are more detrimental as they mostly contain the pharmaceuticals in their most raw form, without dilution. However, they can be better managed through proper waste collection, transportation, and disposal of pharmaceuticals.

## Fate and distribution of pharmaceutical drugs

The environmental conditions and the resultant metabolites of the drugs are the major factors that ultimately determine the fate of the drugs. On the other hand, the bioaccumulation, aqueous solubility, biotransformation, bioavailability, photolysis, and the sorption of sediments also determine the fate and distribution of the drugs in water resources and into the environment (Gwenzi et?al., 2022). The prevalence of pharmaceutical drugs was observed in both surface water and sediments by Matongo et?al. (2015), Ncube et?al. (2021), and Xiang et?al. (2021). Also, sediments represent the interaction of the aquatic and terrestrial ecosystems and therefore are a critical component of the distribution of pharmaceutical drugs (Hernando et?al., 2006). This shows the interdependence of the quality of surface water and sediments. The hydrodynamics essentially influence the distribution of the pharmaceuticals in water resources, because of their different physicochemical properties (Ncube et?al., 2021). Xu et?al. (2019) observed a correlation between hydrodynamic residence and the contamination concentration of pharmaceutical drugs in water resources because of their static hydrodynamic flushing. This is because the sufficient flushing of contaminants in a water resource requires relatively low contamination concentration and hydrodynamic residence. Therefore, high contamination and hydrodynamic residence impede sufficient flushing in water resources, thus increasing the distribution and availability of pharmaceutical drugs.

Another influence on the fate and distribution of pharmaceutical drugs is the physicochemical characteristics of the drugs (Matongo et?al., 2015). As shown on Table 1, the physicochemical characteristics of the four target drugs for this current work are demonstrated. Most pharmaceuticals are characterised by long surface water longevity, and this causes the pharmaceuticals to be more persistent and have a longer dissemination period (Xu et?al., 2019). Carbamazepine is consistently detected in most surface water globally due to its long half-life estimated at around 900 h (Waleng & Nomngongo, 2022; Xiang et?al., 2021). This observation makes carbamazepine to accumulate and distribute significantly in water resources. However, pharmaceuticals with shorter half-life in surface water still disseminate significantly due to their constant release into surface water resources; thus, making such pharmaceuticals pseudo-persistent pharmaceutical pollutants. For example, ibuprofen was still detected in high concentrations in the coastal water samples of Singapore, despite its short half-life in surface water and being one of the few readily biodegradable pharmaceuticals (Waleng & Nomngongo, 2022). This indicated the persistence of short half-life pharmaceutical drugs in surface water due to their constant release. This could be the reason for the persistence of some of the Covid-19 drugs in water resources and the environment. Also, the half-life periods for the four target drugs for this study in water resources needs to be determined from multiple studies for advancement in the understanding of the drugs. Again, pharmaceuticals need to be managed from their sources to reduce their distribution concentration in water resources and on the environment.

**Table 1:** Physicochemical characteristics of the four Covid-19 target drugs

Physicochemical Characteristics	Azithromycin	Prednisone	Prednisolone	Dexamethasone	References
Molecular structure					Morales-Paredes et al. (2022); Pan and Yau (2021); Gong et al. (2019); He et al. (2022); Inarmal and Moodley (2023)
Molecular weight (g/mol^?1^)	785.0	358.4	360.4	392.4	Morales-Paredes et al. (2022); Pan and Yau (2021); Gong et al. (2019); He et al. (2022); Inarmal and Moodley (2023)
Solubility in water at 25 °C	0.1 mg/mL	0.133 mg/mL	223 mg/L	_	Morales-Paredes et al. (2022); Pan and Yau (2021); Gong et al. (2019); He et al. (2022); Inarmal and Moodley (2023)
Octanol?water partition coefficient (Log Kow)	4.32	1.62	_	1.83	Morales-Paredes et al. (2022); Pan and Yau (2021); Gong et al. (2019); He et al. (2022); Inarmal and Moodley (2023)

### *Pseudo*-partitioning and leaching assessment for fate determination of Covid-19 drugs

There is a need for a broader understanding of the fate of Covid-19 drugs in water resources as they are currently emerging contaminants of concern. Therefore, it is pivotal to delve into the nature of how the Covid-19 drugs behave in both solid and liquid phases in water resources. In the case of water resources, the P-?PC can be determined to attain a quantitative knowledge of pharmaceuticals in the water and sediment phases. On the other hand, it is essential to understand the leaching potential of Covid-19 drugs, which will help assist in understanding their concentration in surface water resources and sediments, thus posing a critical threat to pollution of the groundwater (Pei et?al., 2022). For this leaching assessment, the groundwater ubiquity score method can be applied to the Covid-19 drugs in an attempt to determine their leaching potential (Pei et?al., 2022). Pei et?al. (2022) applied these methods in the study to assess the behaviour of antibiotics and personal care products in water resources and sediments. However, there is no evidence in the literature where P-PC and the groundwater ubiquity score were determined for Covid-19 drugs. Such is critical for the management of environmental surface water resources by significantly contributing to the understanding of the fate of Covid-19 drugs in water resources.

## Occurrence of potential harmful pharmaceuticals in wastewater effluents and water resources

### Occurrence of azithromycin in wastewater effluents and water resources

Azithromycin is ranked the third most consumed antibiotic drug because of its immunomodulatory and antiviral effects (Mirzaie et?al., 2022). Because of its constant use and high consumption, this makes azithromycin a pseudo-persistent contaminant (Pan & Yau, 2021). Therefore, traces of azithromycin have been found in wastewater effluents and surface water resources. Morales-Paredes et?al. (2022) reported concentrations of azithromycin of about 935 ng/L in surface water in Spain. These surface waters contained treated effluent from domestic WWTFs that had a rise of about 217 times of azithromycin concentrations during the peak period of the Covid-19 pandemic. The study further indicated that azithromycin metabolites are contained in the sediments of the tested surface water resources. In line with this, Mirzaie et?al. (2022) investigated possible pollution by azithromycin in the Persian Gulf Sea in West Asia. The findings indicated pollution by wastewater effluent that contained a concentration of azithromycin of about 896 ng/L. Furthermore, the sediments in the sea contained azithromycin concentrations of between six and nine?nanograms per litre. The study of Pan and Yau (2021) also presented an abundance of azithromycin concentrations from wastewater effluent, ranging from 571 ng/L to 1?046 ng/L. This study was done in China on five WWTFs. This also presented azithromycin as one of the most used antibiotics globally and its prevalence in wastewater effluent post the Covid-19 pandemic. These studies showed high occurrences of azithromycin in wastewater effluents and surface water resources. This has the potential to lead to a high risk of toxicity on aquatic ecosystems and human health as freshwater resources are vital for the survival of humans and animals.

South Africa is no exception to the pollution of environmental water by pharmaceutical drugs. Some of the research done in the country observed pharmaceutical pollution in environmental waters. The Umgeni River in the KwaZulu-Natal province was tested for the prevalence of antibiotics, including azithromycin. The results showed a high prevalence of antibiotics with concentrations exceeding 10 ?g/L on the water and sediments (Matongo et?al., 2015; Ncube et?al., 2021). High concentrations of antibiotics of a maximum of 8.27 ?g/L were observed by Lehutso et?al. (2017) on water samples of a river in the Gauteng province. To add to this, freshwater sources of the Eastern Cape province were observed to have traces of antibiotics, thus leading to a public health threat as such water is used for consumption and domestic activities (Vumazonke et?al., 2020). Also, these studies illustrated that although there is limited research on pharmaceutical pollution in rural areas, two of the top three most rural provinces in South Africa, namely the Eastern Cape and KwaZulu-Natal, indicated evidence of pharmaceutical pollution. Again, Munzhelele et?al. (2024) similarly observed high concentrations of pharmaceuticals in water resources of the Gauteng and KwaZulu-Natal provinces. However, the study did not consider Covid-19 drugs. There is limited information on the distribution of pharmaceuticals including Covid-19 drugs in South African water resources, particularly in rural provinces. This is a detrimental health threat to the vast communities in the rural provinces as they mostly depend on river water for domestic use, including drinking water.

### Occurrence of prednisone in wastewater effluents and water resources

Prednisone is one of the few drugs used to treat Covid-19, with high detection levels in wastewater effluents and environmental water (Gong et?al., 2019). Thus, as prednisone accumulates in environmental compartments like water resources, it poses a human and aquatic health risk. A study by Yazdan et?al. (2021) demonstrated that prednisone is present in surface water at concentrations of about 0.05?433 ng/L, with higher concentrations in surface water that receives treated effluent from WWTFs. This demonstrated limited removal efficiency by WWTFs during treatment and varying concentrations, which could depend on community size and the spread of Covid-19. Countries such as China, Spain, Japan, France, as well as the Netherlands, were found to have concentrations of prednisone ranging between 0.03 and 29 ng/L, with a maximum observed concentration of 230 ng/L in surface water (Gong et?al., 2019). This demonstrated the widespread use of prednisone and possible accumulation and invasion into the natural environment over time due to constant release and accumulation into the environmental water. There is a lack of an exclusive study that investigates and presents the prevalence and distribution of prednisone in African water resources.

### Occurrence of prednisolone in wastewater effluents and water resources

Prednisolone has been detected in various wastewater effluents and water resources across the globe due to its frequent use, particularly for the treatment of Covid-19. This has led to an abundance of prednisolone traces in wastewater effluents and water resources, as it is characterised by its critically low solubility in water (Yazdan et?al., 2021). This also highlights that prednisolone could be found in high concentrations in sediments due to its insolubility in water. A study by Chang et?al. (2007), which assessed the occurrence of six glucocorticoids including prednisolone in WWTFs, demonstrated that prednisolone had the lowest removal rate of all the studied drugs. This was attributed to its low biodegradability, which consistently had an average concentration ranging between 0.56 and 0.66 ng/L. Yazdan et?al. (2021) similarly observed low biodegradability of prednisolone in the water and the environmental compartments. This indicates a fatal risk of the bioaccumulation of prednisolone in aquatic animals and the sediments of water resources. Additionally, Inarmal and Moodley (2023) reported prednisolone at concentrations ranging from 0.008775 mg/L to 0.4482 mg/L in wastewater effluents of South Africa. By far, this is the only study to report on prednisolone traces in wastewater effluent after the Covid-19 outbreak. This is a demonstration that there is little information and knowledge on prednisolone and the recent influence of Covid-19 on the prednisolone concentrations in wastewater effluents and water resources. This poses a potential risk of biomagnification of the drug into the aquatic ecosystem and also in humans.

### Occurrence of dexamethasone in wastewater effluents and water resources

To remedy the effects of Covid-19 on patients, dexamethasone has been used due to its low cost and widespread availability (He et?al., 2022). For this reason, the increased use has led to its presence in wastewater effluents and water resources due to human excretion. Musee et?al. (2021) described dexamethasone as a persistent drug in the WWTFs, thus resulting in its traces in water resources because of inefficient removal. However, the study also indicated that dexamethasone was commonly detected in low concentrations of about six per cent in wastewater effluents and water resources. This could be attributed to the fact that dexamethasone was only used for the treatment of hospitalised Covid-19 patients who were in a critical state and were also on ventilators (Musee et?al., 2021). A study by Hayden et?al. (2022) on effluent in the Penn State WWTF confirmed this assertion by reporting that the concentration of dexamethasone increased exponentially as the number of hospitalised patients increased. This study indicated a direct correlation between increased dexamethasone use with the hospitalisation of Covid-19 patients. Cronin et?al. (2012) observed that dexamethasone has a half-life period of about 36?72 h. This stipulated that dexamethasone excretion from the human body occurs for about three days after treatment. This assertion then stipulates that the presence of dexamethasone in wastewater would occur for longer periods due to continuous excretion by both current and previous patients. Likewise, a study done on an urban WWTF in China observed concentrations of 0.02?0.09 ng/L of dexamethasone in wastewater effluent being disposed of into water resources (He et?al., 2022). This indicates the failure of the removal of dexamethasone from wastewater and the continuous pollution of water resources by this pharmaceutical pollutant.

Although dexamethasone has been detected mostly in low concentrations in wastewater effluents and water resources (Hayden et?al., 2022; Kuroda et?al., 2021; Musee et?al., 2021), its presence and potential accumulation in surface water resources cannot be neglected. Numerous studies globally have observed dexamethasone concentrations in surface water. For instance, before the Covid-19 pandemic breakout, surface water of Hungary, Malaysia, and China observed dexamethasone concentrations of 0.07 ng/L, 0.73 ng/L, and 0.11 ng/L to 0.33 ng/L, respectively (Gong et?al., 2019; Praveena et?al., 2018; Tölgyesi et?al., 2010). Again, post-Covid-19, in Switzerland, Portugal, and Spain, dexamethasone was detected in concentrations of 147 ng/L, 352 ng/L, and 360 ng/L, respectively (Musee et?al., 2021). These studies show the prevalence of dexamethasone in surface water, and it can also be observed that the studies done pre-Covid-19 reported lower concentrations of dexamethasone as compared to those done after the Covid-19 pandemic breakout. This confirms the increased use of dexamethasone as a therapeutic drug for Covid-19 and its increased prevalence in the environment and water resources. In essence, pharmaceutical drugs have persistent properties to the extent that their accumulation in water resources may potentially contaminate the environment, food chain, and water cycle, thus resulting in potential health risks to humans and other living organisms (Medema et?al., 2020; Morales-Paredes et?al., 2022). As shown in Table 2, there is a possible widespread occurrence of Covid-19 drugs in wastewater and water resources. However, there is still minute research that gives a detailed global overview and representation of the occurrence of the Covid-19 drugs, specifically in the sediments of surface water resources and the aquatic life such as fish or algae, as well as in groundwater resources. This is a knowledge limitation and reduces the potential research for determining the associated aquatic and human health risks arising from the Covid-19 drugs in water resources and the environment. It can further be noted in Table 2 that the concentrations of these four Covid-19 drugs increased post the Covid-19 pandemic outbreak in 2020. Studies reported after 2020 present higher concentrations of the Covid-19 drugs than those reported in 2019 and prior. Overall, the current literature findings demonstrate that there is limited global and local diversity in assessing the occurrence of Covid-19 drugs more particularly in water resources.

**Table 2:** Global assessment of selected Covid-19 drugs in wastewater effluent and environmental components

Covid-19 Drug	Location	Wastewater effluent concentration	Environmental component and concentration	References
Azithromycin	Spain	?	Surface water- 935 ng/L	Morales-Paredes et al (2022) Mirzaie et al. (2022) Pan and Yau (2021) Ncube et al. (2021)
	West Asia	896 ng/L	Sediments- 6 ng/L to 9 ng/L	
	China	571 ng/L to 1046 ng/L	?	
	South Africa	?	Surface water- 10 ?g/L	
Prednisone	Globe	?	Surface water- 0.05 ng/L to 433 ng/L	Yazdan et al. (2021)
	China; Spain; Japan; France and Netherlands	?	Surface water- 0.03 ng/L to 29 ng/L	Gong et al. (2019)
Prednisolone	China	0.56 ng/L to 0.66 ng/L	?	Chang et al. (2007) Inarmal and Moodley (2023)
	South Africa	0.008775 mg/L to 0.4482 mg/L	?	
Dexamethasone	China	0.02 ng/L to 0.09 ng/L	?	He et al. (2022) Gong et al. (2019) Praveena et al. (2018) Tölgyesi et al. (2010) Musee et al. (2021) Musee et al. (2021) Musee et al. (2021)
	Hungary	?	Surface water- 0.07 ng/L	
	Malaysia	?	Surface water- 0.73 ng/L	
	China	?	Surface water- 0.11 ng/L to 0.33 ng/L	
	Switzerland	?	Surface water- 147 ng/L	
	Portugal	?	Surface water- 352 ng/L	
	Spain	?	Surface water- 360 ng/L	

## Use of bioassays in determining ecotoxicity risk assessments

Bioassays have been applied for decades to test for ecotoxicity of a variety of chemicals in water resources and wastewater effluents. This procedure exploits living organisms by exposing the organisms to different concentrations of the possible toxic chemical to determine the toxicity of chemicals in water (Hassan et?al., 2016). Moreso, the toxicity of combined chemicals can be determined from this procedure. After the exposure to the chemical, the biological integrity of the organisms is assessed and compared to the organism that was not exposed to the chemical (Musee et?al., 2021). The changes are then recorded and the target organs for the chemicals are also identified. This demonstrates the toxicity of the chemical, and the diseases that could be associated due to the specified target organs.

### Fish bioassays

The use of fish for toxicity assessment has been applied for decades. This is because of the reaction or change of the physiology and behaviour of fish to toxins in water (Hassan et?al., 2016). The lethality on fish is measured by the change in growth, accumulation of the chemical in fish organs, as well as the disturbance in reproduction (Kuroda et?al., 2021; Vestel et?al., 2016). Fish species such as zebra fish, fathead minnow, bow trout, red killifish, common carp, and catfish are primarily the ideal species for toxicity assessments (Hassan et?al., 2016). Fish can be used to assess the toxicity of heavy metals, pharmaceuticals, personal care products, pesticides, herbicides, and other possible chemicals of interest depending on the research focus. Overall, fish can be used to demonstrate the total toxicity effects of chemicals in water resources (Xin et?al., 2020). Fish is abundant in most water resources, and it is easily identifiable, thus it is readily available. Fish is an essential part of the food chain, including human beings (Hassan et?al., 2016). Again, fish have a long life cycle and can thus demonstrate chronic toxicity. However, the downsides of using fish for ecotoxicity tests are that fish have low sensitivity to low concentrations of toxins (Xin et?al., 2020). Additionally, toxicity tests using fish require specialised equipment and skills and take long periods to perform. However, Capela et?al. (2020) and Chahardehi et?al. (2020) have demonstrated that the use of fish embryos is more accurate and can be performed in a short period of time. Again, Capela et?al. (2020) and Chahardehi et?al. (2020) demonstrated that the use of fish embryos reduces animal experimentation, demonstrates the effects of toxins on the fundamental developmental processes, have similar genetic data to humans and other animals, and further fulfils the need for sensitive tools in toxicity testing. Moreso, according to Truong et?al. (2011) fish embryos are produced in large numbers, therefore, they allow for large samples and are then statistically powerful for dose?response studies. Based on these advantages, it is therefore important that the use of fish embryo bioassays is explored for pharmaceutical toxicity testing to demonstrate the effects of pharmaceuticals on human health. Besides the many advantages of fish embryos on toxicity testing, to date, no study has explored fish embryos for toxicity of pharmaceuticals on human health. Therefore, there is a knowledge gap that needs to be filled in terms of the direct human health implications of Covid-19 drugs, and fish embryos are ideal to investigate and fill this knowledge gap.

### Plant and algal bioassays

Plant bioassays, like the use of Chinese cabbage, offer a variety of assessment endpoints that demonstrate the toxicity of specific or combined chemicals. For instance, the plant growth rate, biomass weight, and enzyme activity can be assessed to measure the toxicity of the chemical being assessed (Waleng & Nomngongo, 2022). However, plant ecotoxicity tests require more time, particularly for the measurement of weight and length, and germination scores (Kock et?al., 2023). On the other hand, algae bioassays are also performed for ecological toxicity. This is because algae are sensitive to many substances in water such as chemical compounds and heavy metals (Kuroda et?al., 2021). Therefore, algae can be used to determine the acute or chronic toxicity of a specific toxin being studied.

### Microbial bioassays

Microbial screening tests for toxicity have gained momentum in the last few years. This is because the use of higher organisms such as fish and plants is more expensive and time-consuming. On the contrary, microbial bioassays are inexpensive and have a short life cycle, thus indicating results within a short period. Furthermore, microbial bioassays are sensitive to toxins at very low concentrations (Hassan et?al., 2016). Microbial bioassays are therefore ideal for detecting toxins prevalent in drinking water at minute concentrations. Because of their ability to cover a wide array of toxins, and easy use in the laboratory, microbial bioassays have since been explored and used to represent toxicity on both aquatic life and human health (Kosma et?al., 2014).

## Toxicity and potential risks of pharmaceutical drugs on water resources and the environment

Pollution of water resources and the environment by Covid-19 pharmaceutical drugs has received much attention, particularly during the Covid-19 outbreak in 2020. This is because such pharmaceutical drugs are persistent in the environment due to their half-life period, mobility, transformation products, and metabolites (Gwenzi et?al., 2022; Kuroda et?al., 2021; Morales-Paredes et?al., 2022; Wilkinson et?al., 2022). These characteristics allow the Covid-?19 drugs to accumulate in water resources as well as the environment and lead to their toxicity and potential risk threat to aquatic life, and more crucial, human health.

### Water resources degradation and water resources scarcity

Surface water resources, in particular, are the primary sites that receive pharmaceutically polluted water. The concentration of pharmaceutical drugs is therefore much higher in surface water resources than in groundwater resources (Wilkinson et?al., 2022). This is because surface water resources are more susceptible to the environment. The constant release of wastewater effluent, runoff from agricultural sites and waste disposal sites, leachates from landfills, and raw sewage leaks simultaneously contribute to the significant pollution of water resources by pharmaceuticals (Kuroda et?al., 2021). Dams, streams, rivers, estuaries, oceans, and groundwater globally contain traces of pharmaceutical drugs because of their surrounding land uses, and the mismanagement of waste (Wilkinson et?al., 2022). It has become notable from several recent studies that water resources are gradually exhausting their assimilative capacity (Hayden et?al., 2022; Gwenzi et?al., 2022; Niang et?al., 2023; Waleng & Nomngongo, 2022; Wilkinson et?al., 2022. For this reason, much higher concentrations of pharmaceuticals are consistently observed in water resources. This implies higher pharmaceutical concentrations being received by the water resources, as well as minimal flushing of the water resources. This is an alarming finding that may lead to limited access to freshwater in the foreseeable future.

Asian countries have also experienced serious challenges in terms of pharmaceutical pollution of water resources. For instance, the assessment of surface water in Asia indicated the prevalence of pharmaceutical drug traces (Waleng & Nomngongo, 2022). The concentrations were found to record a maximum of 365.05 ?g/L on samples analysed from surface water. Also, the same study showed that African countries like Kenya, Nigeria, and Mozambique indicated the persistence and prevalence of pharmaceutical drugs in their environmental water samples. Concentrations of pharmaceutical drugs ranging from 53.8 ?g/L to 56.6 ?g/L were detected in the surface water samples. The likelihood of an increase in the concentrations of these pharmaceutical drugs in water resources is eminent. This is primarily driven by more access to health care and the overdependence on pharmaceuticals to treat diseases. As a result, with time, the pharmaceutical pollution of water resources can pose a toxicity threat to aquatic life, wildlife, and human health.

### Degradation of aquatic organisms

The aquatic life is dependent on the water quality of the water resources that serve as their natural habitat. Since aquatic organisms spend most of their life cycle in these water resources, they are at risk of taking up and storing these pharmaceutical drugs in their tissues (Kuroda et?al., 2021). Certain fish and algae that thrive in pharmaceutically polluted water have been known to accumulate such drugs in their tissues. For the most part, fish are used primarily for toxicity studies because of their notable behaviour and physiology (Waleng & Nomngongo, 2022). As an example, the body length and embryos of the zebra fish have been documented to potentially reduce in size and quality due to exposure to pharmaceutical drugs in oceans (Musee et?al., 2021). This indicated the need to reduce the contamination of oceans by pharmaceutical drugs to protect the quality of the zebra fish species. In addition, Vestel et?al. (2016) denoted that the exposure of fish species to pharmaceutical drugs could lead to the destruction of their normal reproductive cycle by suppressing ovulation. This can be regarded as a possible extinction threat to many fish species. This study also indicated the possible hormonal imbalances in aquatic animals due to their exposure to pharmaceutical drugs, specifically for corticosteroids such as dexamethasone, prednisone, and prednisolone drugs that are deleterious to the ecosystem as they deter the growth and reproductive processes of fish. Musee et?al. (2021) observed a reduction in fish population, hormonal imbalances, and smaller gonads, but more critical effects such as cancer or death have not been observed yet. On the other hand, Allouche et al. (2022) observed the reduction in body size and impaired functional changes in meiobenthic nematodes after exposure to dexamethasone and prednisolone at laboratory scale for 30 days. Similarly, Mhadhbi et al. (2022) observed larvae mortality as well as morphological abnormalities in the early life stages of the European sea bass (*dicentrarchus labrax*) after exposure to different azithromycin concentrations at laboratory scale. The results further revealed functional impairments of the liver and gills of the *dicentrarchus labrax.* Again, Rutkoski et al. (2024) reported changes in thyroid glands, reduction in diameter as well as the number of follicles and increased oxidative stress in *aquarana catesbeianus* tadpoles. These tadpoles were exposed to prednisone and prednisolone at environmentally friendly concentrations of 0.01, 1 and 10 ?g/L in the laboratory. These studies demonstrate the toxicity and lethality of Covid-19 drugs in water resources to macroinvertebrates, therefore, more experiments are required to improve knowledge on the risks and toxicity of the Covid-19 drugs due to water resources. Also, the recurring use of such drugs, and increased environmental concentrations indicate the potential for the development of more adverse effects over the long-term.

Different algae species are also capable of absorbing pharmaceutical drugs into their thallus. Moreso, algae form the foundation of the food chain in the aquatic ecosystem and water purification (Vestel et?al., 2016). In essence, the quality of algae is essential for ecosystem services rendered by water resources (Hayden et?al., 2022; Vestel et?al., 2016). A study by Kock et?al. (2023) observed that marine algae are most sensitive to antibiotic exposure. This is because the antibiotics affect the photosynthetic and chloroplast components of algae, thus inhibiting the primary function of the algae. Consequently, this affects the composition of the algae community and ultimately interrupts the primary production of algae. Correspondingly, Kosma et?al. (2014) reported that algae seemed to be the most sensitive species to pharmaceutical pollution in water resources due to the observed accumulation of pharmaceuticals in the thallus of the algae. These studies indicated that algae are negatively impacted by pharmaceutical pollution, and this may be due to its inherent nature of purifying water in water resources, thus algae absorb and accumulates more pharmaceuticals.

Diatoms form an essential integral part of the aquatic ecosystem as they are microalgae. However, the introduction of pharmaceutical drugs into the environment has potentially impacted their quality and population (Kock et?al., 2023). Therefore, the observed impacts may be used as an indicator of pharmaceutically induced impacts on higher trophic level organisms and the ecosystem at large. Diatoms are used for ecotoxicological assessment of water resources because of their ubiquity and responsiveness to sudden or overtime changes in water resource environments (Kergoat et?al., 2021). Being a vital source of food to primary consumers, this makes diatoms a critical link to higher trophic species (Kergoat et?al., 2021; Kock et?al., 2023). This assertion demonstrates the possibility of progressive pharmaceutical pollution of the numerous trophic levels. Diatoms are further characterised by limited mobility and can therefore indicate pollution of a specific area in a water resource (Xin et?al., 2020). As an example, Robson et?al. (2020) have observed that diatom assemblages when exposed to pharmaceutical drugs in water resource environments, shift significantly in their community structure. This has been justified by an observation of varying populations with less population in polluted areas. Similarly, Kergoat et?al. (2021) detected the loss of diatom richness and population in water resources due to pharmaceutical pollution. Therefore, this implies that diatoms are negatively impacted by pharmaceutical pollution on water resources, and this may lead to population loss and detrimental destruction to the food chain. Also, diatoms can play an important role in identifying sections of a polluted surface water resource. Therefore, diatoms can be used as bioindicators for pharmaceutical pollution in water resources.

### Degradation of human health

The abundance of Covid-19 antibiotic pharmaceuticals in water resources has the potential to enhance the development of antibiotic-resistant genes and bacteria. As a result, the World Health Organization has prioritised antibiotic resistance and placed it among the top 10 public health threats globally (cited by Kock et?al., 2023). This is alarming as it can be projected that in the near future antibiotics may be ineffective in the treatment of human and animal diseases or infections. This will cause the severe spread of infections and possibly high human and animal mortality. WWTFs are the main receptors of waste that contain high antibiotic concentrations (Wilkinson et?al., 2022). This implies that wastewater effluents that are released into water resources and the environment are the primary sources of antibiotic-resistant genes and bacteria.

The continuous use and spread of antibiotic azithromycin intensify the pollution threat and the recurring development of azithromycin-resistant genes and bacteria. Munzhelele et?al. (2024) confirmed the spread of antibiotic resistance in water resources and the environment being attributed to wastewater effluents. Bengtsson-Palme et?al. (2021) demonstrated that wastewater effluents that contain significantly high concentrations of azithromycin contain high levels of azithromycin-resistant genes and bacteria. This antibiotic has been found in many WWTFs and water resources globally (Wang et?al., 2023). This shows the significant spread of azithromycin-resistant bacteria and further implies that azithromycin may not be effective in treating Covid-19 and other diseases in the future if such spread of azithromycin-resistant bacteria is not controlled effectively. To add to this, Mirzaie et?al. (2022) showed that the increased use of azithromycin will enhance the growth of azithromycin-resistant bacteria and genes and this phenomenon will be observed long after the Covid-19 pandemic.

Studies have demonstrated the prevalence of azithromycin in drinking water sources from taps and wells (Ben et?al., 2020; Wang et?al., 2023). On the other hand, researchers have only demonstrated the side effects and effects of long-term use of azithromycin such as vomiting, nausea, abdominal pain, and more critically, liver damage (Mirzaie et?al., 2022). However, to date, there is no knowledge of the long-term human health effects of exposure to azithromycin by direct use of surface water. There is a need for an extension of such knowledge.

### Receptors of Covid-19 pharmaceutical drugs

#### Water resources

Water resources face the detrimental challenge of pharmaceutical pollution as they are the primary disposal sites for most wastewater effluent (Maremane et?al., 2024). This leads to the accumulation of pharmaceutical drugs and their metabolites in water resources. The most recent global study of pharmaceutical pollution on water resources, which focused on 258 rivers in 104 countries, representing all continents, observed significant global surface water pharmaceutical pollution (Wilkinson et?al., 2022). This study of Wilkinson et al. (2022) demonstrated a wide spread environmental pollution by pharmaceuticals and therefore highlights the urgent need to also assess the prevalence of Covid-19 drugs in the environment and water resources. The study also found that the highest cumulative active pharmaceutical ingredients were observed in rivers of South Asia, sub-Saharan Africa, and South America. This indicated that most pharmaceutical contamination of surface water occurred in low- to middle-income countries. This could be attributed to inefficient wastewater management, poor waste management infrastructure, and pharmaceutical manufacturing inadequacies. In addition, the study found that the amount of research data on pharmaceutical pollution of rivers in Africa was limited; the majority of available research data came from Northern America and Western Europe. Therefore, there is a need for an intensive study on pharmaceutical pollution in African rivers, including South Africa.

Asian and African regions have a huge gap in data on the occurrence and distribution of pharmaceuticals in water resources. For instance, a study conducted in Asia and Africa revealed that Asia only has minute studies on the occurrence of pharmaceuticals in water resources (Waleng & Nomngongo, 2022). Yet, the Asian Health Institute has reported a tremendous rise in the consumption of most pharmaceuticals, particularly during the peak period of the Covid-19 pandemic. This indicated a very limited assessment of water resources for pharmaceutical pollution in Asia. In South Africa, Waleng and Nomngongo (2022) showed that a river in the Gauteng province indicated high concentrations of a variety of pharmaceuticals. Again, the Umgeni River in the KwaZulu-Natal province detected relatively high amounts of pharmaceuticals in the river water and sediments, which were recorded at above 10 ng/L. This showed that there is a possibility of high pollution by pharmaceutical drugs in South African water resources. However, not all surface water from various provinces of the country has been assessed. In actuality, a large percentage of the country?s water resources have been excluded from pharmaceutical pollution assessments or research. To counteract this assertion, there is a need for a study that assesses multiple water resources for pharmaceutical pollution to ascertain the extent of pollution and geographical distribution of pharmaceutical pollutants in the context of South Africa. This calls for progressive research in South Africa to assess the occurrence and fate of pharmaceutical drugs in water resources.

#### Humans

Water is a source of life, and all living things depend on water. Therefore, the pollution of water by pharmaceuticals directly leads to the consumption of contaminated water by living organisms, including humans (Kock et?al., 2023). Human beings utilise and consume water from both surface water and groundwater sources. Although drinking water is treated to safe standards for human consumption, the conventional treatment of drinking water does not remove pharmaceuticals from the water. Thus, humans consume the pharmaceutically contaminated water. A study by Bexfield et?al. (2019) in the United States found considerably high traces of pharmaceuticals in groundwater sources that were used for domestic purposes, including human consumption. This then indicated the vulnerability of groundwater to pharmaceutical contamination and also a direct exposure to pharmaceutical drugs to humans. In addition, another study reported by Wang et?al. (2023) detected pharmaceuticals, which were mostly antibiotics, in drinking water from taps and wells in Eastern China. This is an indication that should heighten the possibility of long-term effects of pharmaceutical consumption by humans. An overexposure to antibiotics in humans may result in antibiotic resistance, gut microbiota changes, gastrointestinal issues and more severely, premature deaths (Kock et?al., 2023; Wang et?al., 2023).

South Africa also faces detrimental challenges of the pollution of freshwater resources by pharmaceuticals and the traces of pharmaceuticals in drinking water. According to Atangana and Oberholster (2023), countries in the sub-Saharan region will have limited access to drinking water due to high contamination of freshwater sources. A study by Verlicchi and Grillini (2020) observed significantly deteriorating surface water and groundwater quality in South Africa and Mozambique due to contamination by pharmaceuticals. This study further observed that the most significant contamination of surface water that affected drinking water quality was predominately observed in the rural and peri-urban areas of South Africa and Mozambique. This can be attributed to poor sanitation and inadequate management of waste in rural areas of the two countries. This also indicates a critical human health risk to rural communities due to heavily contaminated sources of drinking water. Moreover, drinking water sources such as dams, rivers, and groundwater of most parts of the Eastern Cape province in South Africa were observed to have high concentrations of pharmaceuticals, particularly antibiotics (Vumazonke et?al., 2020). This further indicates the probability of poor drinking water quality due to limited access to advanced treatment methods in South Africa.

Covid-19 drugs pose a variety of human health risks that are detrimental and could affect the functionality and life span of humans. To adduce this, Wang et al. (2023) demonstrated that azithromycin can negatively affect the human gastrointestinal tract. This results in nausea, vomiting, and in severe cases, stomach and colon cancer. On the other hand, dexamethasone was found to cause recurring allergic infections, heart damage and also eye problems such as glaucoma in humans (Ben et al., 2020). Additionally, prednisone and prednisolone were reported to cause retention of sodium and fluid in human cells, high blood pressure, headaches, and muscle weakness (Mirzaie et al., 2022). It can be noted that these human health implications of Covid-19 drugs are common health problems in the elderly population. Therefore, a more precise study that directly correlates such health complications to the long-term exposure of humans to Covid-19 drugs in water resources is needed. Such information will undoubtedly demonstrate the dire health risks of Covid-19 drugs to humans through water resources. Therefore, this calls for the assessment of more water resources in South Africa for pharmaceutical pollution to make informed decisions on how to manage the water quality of surface water and groundwater resources, particularly those that are used for drinking water purposes. In essence, humans, as the receptors of pharmaceutical drugs from water resources are at great risk of developing the possible human health effects of pharmaceuticals in the long-term.

#### Aquatic animals

**Fig. 1:**
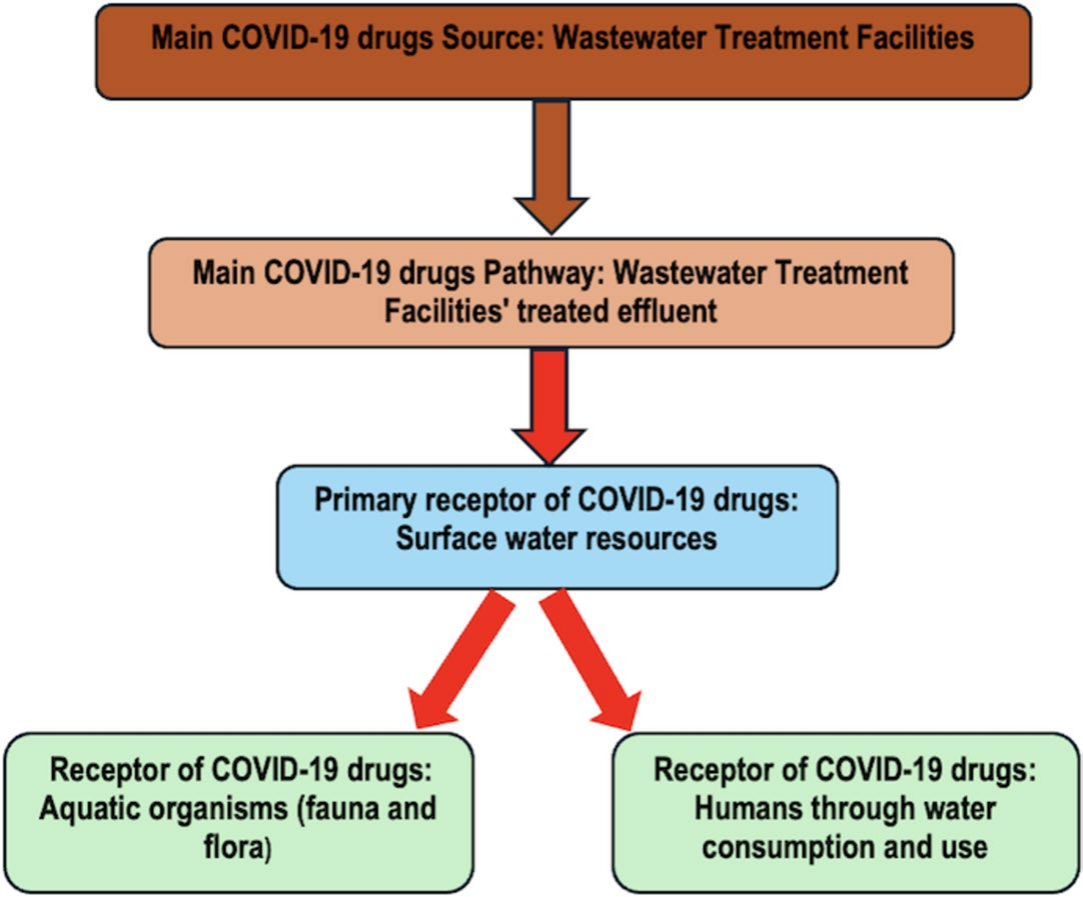
Typical source, pathway, and receptor route for Covid-19 drugs (Authors? own, 2024)

The direct use of pharmaceutically polluted water has a negative impact on the health and functioning of aquatic animals. Most aquatic animals are sensitive to drugs that may hinder their functions and potentially lead to their extinction. For example, the prolonged exposure of pharmaceutical drugs to fish in water resources has led to the presence of such pharmaceutical compounds in several tissues of the fish, such as the brain, liver, muscles, gills, and blood plasma (Waleng & Nomngongo, 2022). Likewise, a Colombian study also observed the accumulation of pharmaceutical drugs in fish tissue, which was due to the presence of such drugs in the surface water that was the habitat of the fish (Pemberthy et?al., 2020). These studies, essentially, showed that aquatic animals such as fish are the receptors of pharmaceutical drugs and their metabolites that end up in surface water resources. For this reason, this directly impacts humans as they consume fish, thus leading to biomagnification because of the pollution of various trophic levels of the ecosystem. Figure 1 illustrates the typical route of pharmaceuticals from source to receptor from an environmental and water resources perspective.

## Assessment approaches of potential harmful pharmaceuticals in water resources

Assessment approaches of pharmaceuticals in water resources are necessary to demonstrate the extent of the lethality of pharmaceuticals and an overview of the extent of pollution. Different researchers have used the risk quotient (RQ) parameter to assess the level of pharmaceutical risk in a variety of environmental media such as soil, water, animals, and plants (Hayden et?al., 2022). Similarly, other researchers have used the scenario evaluation-based approach (Bercu et?al., 2008; Kumar et?al., 2010), and also the exposure-related parameter approach to delve into the consequences of environmental exposure to pharmaceuticals (Kumar et?al., 2010). Water resource pollution risk indices are gaining momentum by being used regularly to assess the level of pollution on water resources, together with the risks that may arise from the pollution of the water resources by pharmaceuticals (Hayden et?al., 2022; Kosma et?al., 2014; Nieto-Juárez et?al., 2021).

### Use of water resource pollution risk indices to assess water and environmental pollution and the risks arising from such pollution

The RQ parameter is determined to assess the risk or hazard posed by a specific pharmaceutical compound on a water resource or the environment. This will help to grade or classify the possible risks to the aquatic and environmental ecosystems. This RQ parameter was developed according to the European Communities (Water Policy) Regulations (2003). These risk assessment indices follow the basic principles of comparing the measured environmental concentrations or the predicted environmental concentration of a given target compound against the predicted no effect concentration, which represents no effect on exposed organisms to the pharmaceutical compound (Nieto-Juárez et?al., 2021). These indices have been applied in various studies to assess the possible ecotoxicity of pharmaceutical drugs. By way of illustration, Nieto-Juárez et?al. (2021) did a study on the environmental risk assessment of pharmaceuticals in the rivers and WWTFs of Peru. The study observed that in the river, antibiotics such as azithromycin, posed a high environmental risk. This was determined by an RQ???1, which indicated the persistence and accumulation of antibiotics in the rivers of Peru. Furthermore, Hayden et?al. (2022) assessed the ecotoxicity of pharmaceuticals, including dexamethasone, on fish, algae, and daphnia in central Pennsylvania. The study revealed that pharmaceutical drugs had a low to moderate risk quotient (RQ???1) on the environmental compartments assessed. Although this showed no critical risk to water resources and the environment, the cumulative impacts of individual pharmaceutical drugs on water resources and the environment over time cannot be neglected. This is because of the constant release of pharmaceuticals into water resources and the environment. Additionally, Kosma et?al. (2014) assessed the impacts of pharmaceuticals on water resources in Greece, and the results revealed that most of the pharmaceutical drugs had an RQ???1 that showed a critical environmental ecotoxicology. This showed the need for better management of pharmaceutical drug pollutants on water resources and the environment. Overall, these studies showed the importance of applying the risk quotient indices to assess the ecotoxicity of pharmaceutical drugs on water resources and the environment. This approach is important as it provides information about the possible state, level of pollution, and also possible future projections.

### Human health risk assessment

Human health risk assessments have presented a challenge in terms of the measure of toxicity of pharmaceuticals in water resources and the environment and how they affect human health. This is because the concentrations observed in water resources do not present as harmful levels to human health (Kumar et?al., 2010). Nevertheless, the scenario evaluation-based approach has been used in various studies such as Bercu et?al. (2008), Kumar et?al. (2010), Rowney et?al. (2009), Schulman et?al. (2002) and Schwab et?al. (2005), and to estimate possible human health risk by the use of a number of assumptions for the developing exposure to a certain pharmaceutical drug or drugs. This approach is closely similar to the predicted no effect concentration approach used for the assessment of pharmaceuticals in water (Kumar et?al., 2010). However, other factors that may contribute to human health risks, which have not been included in risk assessment of human health, include the inhalation of pharmaceuticals when applying reclaimed water and biosolids that are pharmaceutically contaminated (Tarazona et?al., 2021). As well as the ingestion of food and crops grown on soil that was watered and conditioned with pharmaceutically polluted biosolids, and lastly, dermal exposure (Vestel et?al., 2016). These are also pathways that potentially expose humans to the risk of pharmaceuticals and need attention in research when assessing the possible risk on human health.

On the other hand, an exposure-related parameter approach has also been applied to potentially assess the human health risk of pharmaceutical drugs in water (Kumar et?al., 2010). The approach uses scenario-specific information during the process of identifying hazards. In the absence of exposure-related data, assumptions were made for exposure frequency, pharmaceutical concentrations, and exposure duration (Kumar et?al., 2010). By way of illustration, studies have used the pharmaceutical concentration of surface water to estimate the potential risk of a specific pharmaceutical product on human beings (Cunningham et?al., 2009; Schwab et?al., 2005; Webb et?al., 2003). This was based on the assumption that the drinking water treatment facilities do not remove the pharmaceuticals from the raw surface water during treatment. This means that the pharmaceutical concentration was similar to pre-treatment and post-treatment of the drinking water. Although this approach does indicate the potential risk of the pharmaceutical drugs, it excludes the potential effects of the metabolites that may arise as a result of the parent pharmaceutical drug. These metabolites may, however, be more dangerous due to the chemical changes. To adduce this, Kumar et?al. (2010) investigated the removal of a number of pharmaceutical drugs from a drinking water treatment facility, and the results demonstrated that some pharmaceutical drugs were completely removed, whereas else some of the drugs were not even reduced. These findings show that pharmaceutical drugs have different levels of persistence and therefore require to be identified individually and be treated individually for the safety of human health. Therefore, a more specific approach to the human health effects of pharmaceutical drugs in water resources and the environment needs to be developed. The literature, however, shows that to date publications have concluded that environmental risk exposures to pharmaceutical drugs have not affected human health (Medema et?al., 2020; Morales-Paredes et?al., 2022; Singer et?al., 2023). Nevertheless, the literature shows that concentrations of pharmaceuticals are increasing exponentially in the environment and water resources (Hayden et?al., 2022; Kuroda et?al., 2021; Niang et?al., 2023). For this reason, this will cause a human health risk in the coming years due to the accumulation of pharmaceutical drugs in water resources and the environment, thus increasing their concentrations. There is therefore a need for new knowledge of the long-term health risks of pharmaceutical drugs that were exposed to human beings through the environment and water resources in low traces. There is, furthermore, a need to advance these risk assessment approaches and include the long-term risk as well as short term risks of both low and high concentration exposure of pharmaceutical pollutants in water resources. This will clearly outline the associated human health risks of pharmaceuticals in water resources. Additionally, the combined risks of simultaneous exposure of Covid-19 drugs to human health is unknown. There arises a novel need to investigate these combined risks as they may occur due to the simultaneous occurrence of pharmaceuticals in water resources and these combined risks may be more fatal.

## Interactions of pharmaceuticals with heavy metals

Both pharmaceuticals and heavy metals are pollutants of emerging concern exacerbated by the social and economic development of society (Belle et al., 2021; Hayden et al., 2022). Therefore, there are rising concerns as concentrations of these compounds increase in environmental water resources. This causes the possibility of the interaction of pharmaceuticals and heavy metals, thus creating more toxic compounds that have mixed and transformed. Consequently, this threatens aquatic life and human health. There arises a need to monitor individual pharmaceutical drugs and heavy metals, but also the resultant transformed mixed pollutants of pharmaceutical drugs and heavy metals.

### Sources of heavy metals in water resources

Heavy metal contamination presents a danger to all ecosystems, including living organisms and humans. Heavy metals are characterised by their low degradation in the natural environment and their persistence (Aziz et?al., 2023), thus accumulating over time. The key drivers of heavy metal pollution in recent years are mainly urbanisation, industrialisation, expansion in agricultural activities, and climate change (Aziz et?al., 2023; Zhao et?al., 2023). Heavy metals have a high atomic weight and density and can therefore be catastrophic to the environment, water resources, and to human health (Andreu et?al., 2016). Therefore, heavy metals are toxic and can be characterised as carcinogenic, and are known to bioaccumulate in biological and aquatic systems and habitats (Zhao et?al., 2023). In view of this, heavy metals have become a global concern due to their pollution on water resources, thus heightening public concern for human health and the environment. For this reason, it is imperative to understand the sources, leaching processes, chemical transformations, and ways in which heavy metals are deposited into the environment and water resources. Such understanding provides a clear foundation to develop and implement plants for the management of heavy metal pollution.

Natural causes and anthropogenic activities are the primary sources of heavy metal pollution. Among others, human activities such as mining, agricultural practices, and industrialisation, the use of industrial water for irrigation are examples of anthropogenic activities that result in heavy metal pollution (Andreu et?al., 2016; Belle et al., 2024; Zhao et?al., 2023). On the other hand, natural occurrences such as the weathering of rocks, volcanic eruptions, wildfires, and biogenic processes also contribute immensely as sources of heavy metal pollution to the environment and water resources (Kumar et?al., 2010). Sources of heavy metals include wastewater with combined sources of electroplating waste, production of electronic devices, fertilisers, nuclear fuel, and chemical etching (Adeyinka et?al., 2023). By way of illustration, Bakare and Adeyinka (2022) assessed influent and effluent samples from four WWTFs in Durban, KwaZulu-Natal, South Africa. Heavy metals were detected in both influent and effluent samples and all the levels of heavy metals in the effluent samples were non-compliant with the permissible guidelines. To add to this, iron (Fe) had the highest concentration in the effluent. This study demonstrated that WWTFs are also significant contributors to heavy metal pollution on the environment and water resources as they dispose of the effluent into such natural compartments without efficient treatment or removal of the heavy metals. A number of studies have also shown that agricultural practices are the major contributor to considerably high amounts of heavy metals such as arsenic (As), cadmium (Cd), lead (Pb), mercury (Hg), nickel (Ni), and zinc (Zn), (Adeyinka et?al., 2023; Andreu et?al., 2016; Zhao et?al., 2023). Other natural sources that distribute significantly high amounts of heavy metals are atmospheric deposits transported to the earth?s surface by precipitation (Sankhla & Kumar., 2019).

### Prevalence of heavy metals in water resources

Surface water resources are more vulnerable to heavy metal pollution due to the ease of environmental exposure. Therefore, the direct discharge of wastes that contain traces of heavy metals negatively impacts the surface water quality. To illustrate this, a global review study by Sankhla and Kumar (2019) who reviewed 147 publications from 1994 to 2019 on surface water heavy metal contamination, observed that As, Cd, cobalt (Co), chromium (Cr), manganese (Mn), and Ni, were recorded to be above the permissible guidelines in surface water throughout the years. Furthermore, all assessments applied to measure the pollution of heavy metals on the water resources, also indicated dire heavy metal pollution on surface water resources. This showed that in close to three decades, there has been continuous persistence and release of heavy metals into surface water resources, thus leading to heavy metal pollution. Additionally, this shows an abundance of heavy metals in water resources globally. Such an extent of heavy metal pollution will cause a reaction of heavy metals with other pollutants such as pharmaceutical drugs within water resources and the environment. This will lead to the emergence of mixed pollutants that are more toxic. Aziz et?al. (2023) similarly reported heavy metal pollution in river basins of Mexico, China, Iran, Latin America, Ethiopia, Asia, and Bangladesh, with As presenting the highest concentrations and persistence. This has resulted in an abundance of As in the drinking water of Asia, considerably compromising the drinking water quality.

Shared water resources among countries also present the cross-contamination threat of heavy metals and metalloids. As an example, a study by Genthe et?al. (2018) observed that the Olifants River was polluted by heavy metals due to many crocodiles dying on the border of South Africa and Mozambique since 2008. The study then observed heavy metal pollution of the shared water resource in all water samples from South Africa and Mozambique. Of the nine heavy metals assessed against the drinking-water limits of the World Health Organization (2022), eight of them were non-compliant from the South African water samples and only one heavy metal was non-compliant from the Mozambiquan samples. Arsenic had the highest concentration in both countries and further posed a cancer risk to humans consuming water from the catchment. This showed that the source and prevalence of heavy metals was upstream of the Olifants River in South Africa and this may be attributed to heavy mining activities. Thus, the pollution further affected Mozambique downstream of the river.

Groundwater is also polluted by heavy metals that leach through landfill sites (Ulla et al., 2002). These landfill sites further contain medical waste that has pharmaceuticals (Kumar et al., 2010) and these may include Covid-19 drugs. Therefore, it is imperative to invest in the advancement of waste disposal technologies at landfill sites to reduce the leaching of heavy metals as well as pharmaceuticals into groundwater sources. As shown in Table 3, there is global widespread pollution of heavy metals in water resources (Mohankumar et al., 2016; Afzaal et al., 2022; Belle et al., 2021, 2024). This indicates the persistence as well as the accumulation of heavy metals in the environment and water resources. With the current increasing pharmaceutical pollution of water resources (Hayden et al., 2022; Niang et al., 2023; Wilkonson et al., 2022), this is a clear depiction of the simultaneous occurrence of heavy metals and pharmaceuticals. Based on this assertion, it is evident that the development of mixed and transformed pollutants of heavy metals and pharmaceuticals is an occurrence that is likely to emerge soon. Therefore, an intensive research study is needed to determine which heavy metals are likely to bind with pharmaceuticals and also the resultant transformed or mixed pollutants need to be identified. The study should include the primary influences on the bond formation between the heavy metals and the pharmaceuticals. Also, when determining the possibility of mixed pollutants of heavy metals and pharmaceuticals, focus should be given to heavy metals such as As, Al, Fe, Pb, Cu, Cd, Zn, Mg, Mn, and Ni. This is because of their observed recurring occurrence and widespread pollution globally (Afzaal et al., 2022; Aziz et al., 2023; Mutileni et al., 2023; Trisassi et al., 2023). Because of their abundance, such heavy metals are more likely to react with pharmaceuticals in the environment and water resources, then form mixed pollutants.

**Table 3:** Widespread global heavy metal contamination of water resources

Location	Heavy metals	Environmental component	References
Mexico; South Africa; China; Iran; Latin America; Ethiopia; Asia and Bangladesh	As; Al; Cd; Co; Cr; Mn; Mo and Ni	Surface water	Aziz et al. (2023); Sankhla and Kumar (2019); Mollo et al. (2022)
Africa and South Africa	As; Cd; Co; Cr and Pd	Surface water, sediments, and soil	Belle et al. (2024); Yabe et al. (2010)
Pakistan and Egypt	Fe; Mn; Pd; Zn and Pb	Surface water, sediments, and fish	Afzaal et al. (2022); Ghannam (2021)
Pakistan; India; Italy; Iran; Nigeria, Sierra Leon; South Africa	As; Cr; Cu; Al; Fe; Mn; Pb; B; Ni; Mg and Zn	Groundwater	Belle et al. (2021); Ullah et al., (2022); Sankhlar and Kumar (2019); Trisassi et al. (2023); Badeenezhad et al. (2023); Kana (2022); Sankoh et al. (2023); Mutileni et al. (2023); Elumalai et al. (2017); Atangana and Oberholster (2021)
Algeria	Mg and Pb	Spring water and groundwater	Boumaza et al. (2021)
South Africa	Cr; Cd; Fe; Ni; Cu; Hg; Pb and Zn	Sediments and silt	Mohajane and Manjoro (2022); Addo-Bediako et al. (2021); Malidonga et al. (2021); Songca et al. (2013); Edokpayi et al. (2017)

**Fig. 2:**
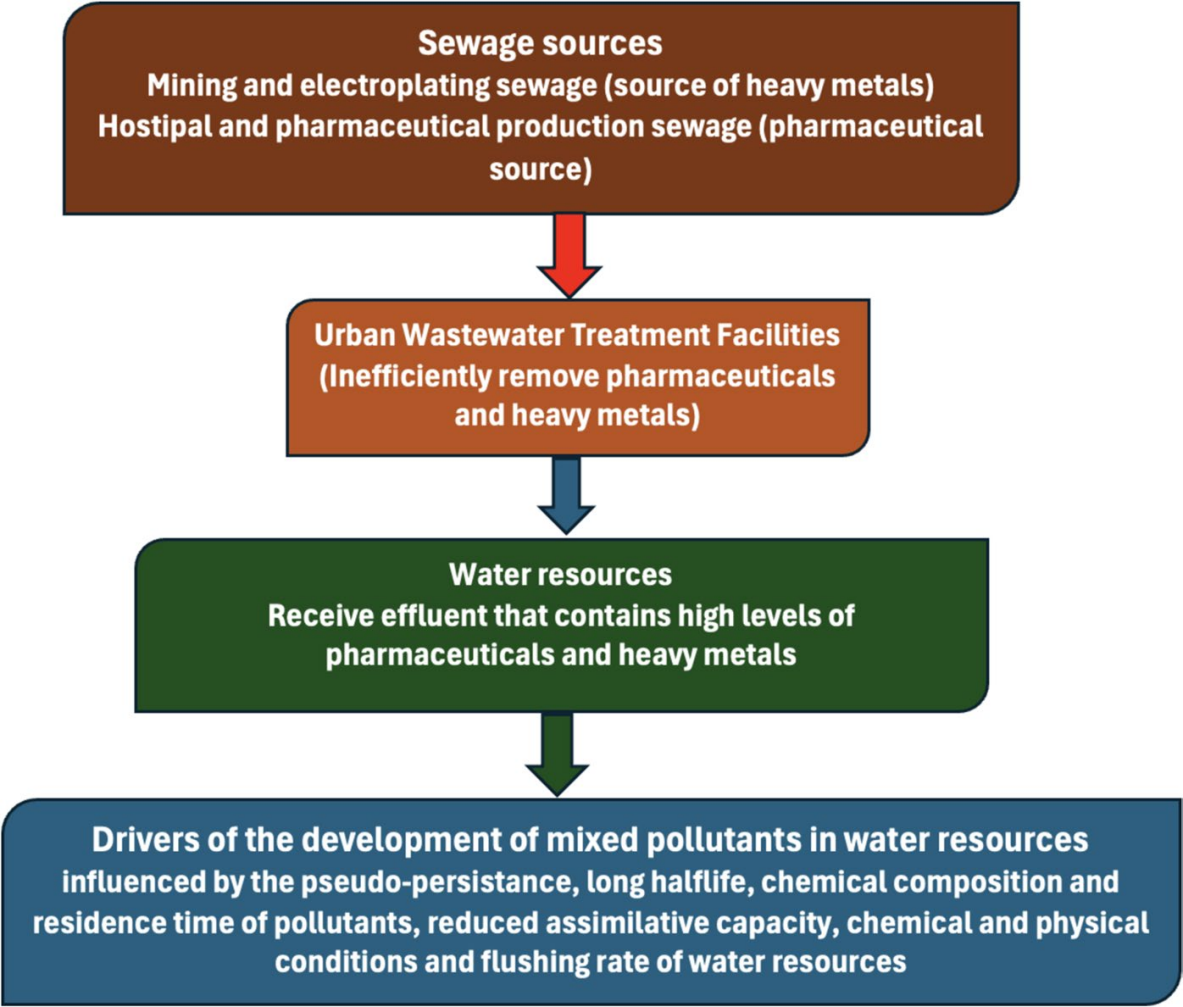
Possible main sources of heavy metals and pharmaceuticals pollutants and the drivers of the development of mixed pollutants of heavy metals and pharmaceuticals in water resources (Authors? own, 2024)

As demonstrated in Fig. 2, the possible development of mixed pollutants could be a result of mining and hospital sewage mixing in urban WWTFs. Because of the limited treatment from WWTFs, the effluent is released with high concentrations of heavy metals and pharmaceuticals into water resources. Due to the constant release of such pollutants, the pollutants continuously react in water resources, transforming and resulting in combined pollutants of heavy metals and pharmaceuticals which to date still require investigation. Such combined pollutants are of undoubted interest and, thus, require an informative detailed study.

### Fate of heavy metals in water resources and sediments

The adsorption?desorption ability of heavy metals determines their bioavailability in water and sediments of water resources. Heavy metals are characterised as non-organic pollutants and therefore they persist in the environment and continuously redistribute in environmental compartments, including water resources and sediments (Arisekar et?al., 2020; Miranda et?al., 2022). Adsorption and desorption are the primary mechanisms by which the interactions between heavy metals with water and sediments are driven. A number of studies have demonstrated that adsorption reactions of heavy metals on sediments result in 90% of the concentrations of heavy metals in urban wastewater on sediments of water resources (Arisekar et?al., 2020; Miranda et?al., 2022; Pandey et?al., 2014). This showed the failure of WWTFs to remove heavy metals during treatment, and also the resistance of heavy metals to basic domestic wastewater treatment technologies. Moreover, the geochemical fractions of the sediments where heavy metals are adsorbed, determine the residence time and residual fractions of the heavy metals in the sediments (Arisekar et?al., 2020; Zhang et?al., 2023). This implies that the metal-sediment interactions can be reduced or reversed depending on specific geochemical conditions of the water resources or sediments. Some environmental factors, such as pH and electrical conductivity (EC), influence the formation and breakdown of chemical bonds between heavy metals and sediments (Miranda et?al., 2022; Pandey et?al., 2014) and therefore determine the desorption and redistribution of heavy metals on sediments into the water. As an example, Miranda et?al. (2022) observed an increase in the solubility of Cr, Ni, and Zn, as a result of the ionic strength of EC in water and sediments. This demonstrated that the concentrations of EC influence the adsorption of the aforementioned metals. However, the study could not determine the relationship between the pH and heavy metals because of the limitations in data sets and the buffering capacity of carbonates from the marine. There is a need to determine the correlation between pH and its influence on the adsorption of heavy metals on sediments. This is because pH is an ion-influenced water variable; thus, it could have an influence on the fate of heavy metals in water and sediments. Given this, the ability of sediments to adsorb heavy metals then regulates the bioavailability and bioaccumulation of heavy metals in water resources and aquatic ecosystems. Additionally, Miranda et?al. (2022) outlined that there is limited knowledge of the role of nutrients like phosphorus and nitrogen on the possible bioavailability of heavy metals in water resources. Furthermore, this demonstrates that the combined effects of the physicochemical properties of sediments and water on the adsorption and desorption of heavy metals remain enigmatic. Heavy metals and pharmaceuticals have similar fate in water resources (Nomngongo & Waleng, 2022; Pandey et al., 2014), this could enhance their interactions to form mixed pollutants. Therefore, this assertion calls for the assessment of heavy metals and pharmaceuticals in water and sediment samples of water resources globally.

## Future prospects and conclusions

Pharmaceutical pollution is a growing concern in water resources globally. The growth in human population and agricultural practises are the primary contributors to pharmaceutical exploitation that leads to the pollution of the environment and water resources. Another contributing factor is the reduced use of indigenous methods to remedy diseases and infections. The same trend has been followed upon the breakout of the Covid-19 pandemic. The literature has demonstrated an abundance of, and a variety of pharmaceuticals, including Covid-19 drugs in wastewater effluents. This is due to their persistence to most wastewater treatment technologies, some are pseudo-persistent, and some have longer half-life periods. There is limited knowledge on the traces of Covid-19 drugs in water resources. Again, researchers have demonstrated traces of some pharmaceuticals in drinking water sources, and this is a critical long-term health risk threat to humans. This is a concern, as Covid-19 drug traces may also emerge in treated drinking water. Studies have observed dire heavy metal pollution globally, and this poses the possibility of a threat of mixed pollutants as pharmaceuticals may react with the heavy metals in water resources resulting in combined pollutants. As a result of the influences of the physical and chemical conditions of water resources, these may primarily drive the interactions of heavy metals and pharmaceuticals such as Covid-19 drugs. Also, inherent conditions such as the chemical structure and composition, residence time of Covid-19 drugs, as well as the flushing rate of water resources may seemingly influence the interaction of heavy metals and pharmaceuticals. A study that compares the influence of each condition on the interactions of heavy metals and pharmaceuticals in water resources is needed. Such information would be pivotal in the management and understanding of the formation of mixed pollutants. Therefore, there is a gap in knowledge in terms of demonstrating the interactions of heavy metals and pharmaceuticals, specifically Covid-19 drugs. Also, the primary drivers of the interactions of heavy metals and pharmaceuticals remain enigmatic and could undoubtedly be of research interest. Because there is no data in the literature that demonstrates combined pollutants of heavy metals and pharmaceuticals, this presents a dire limitation about the knowledge on the fate of pharmaceuticals and heavy metals in water resources. The occurrence, persistence, as well as toxicity of such pollutants, remains enigmatic as it is the most recent research work that has observed this possible phenomenon, but it is yet to be confirmed through novel research.

Most studies have identified the ecotoxicity and risks of pharmaceuticals on aquatic life but have neglected the long-term exposure of such pharmaceuticals to human health. The long-term human and aquatic health risks of pharmaceuticals including Covid-19 drugs from environmental exposure by water resources remain a knowledge gap. There is no known data about the fate and distribution of Covid-19 drugs, and this is a challenge in terms of the management of the pollution of these pharmaceutical contaminants. Also, the present literature overlooked the combined human and aquatic health risks arising from the simultaneous occurrence of Covid-19 drugs as well as the emerging combined pollutants. Such understanding is needed to provide more knowledge on a wider spectrum of risks that may arise from Covid-19 drugs exposure from water resources. There is a handful of literature that explicitly illustrates the synergistic, accumulative, as well as chronic effects of Covid-19 drugs on the aquatic and human health due to exposure to water resources. Such scares knowledge makes conducting human and ecological risk assessments of the Covid-19 drugs a challenge. The influence of nutrients on the bioavailability of heavy metals and Covid-19 drugs has not been investigated; therefore, there is a need to fill such a knowledge gap. More research on pharmaceutical water pollution in low to middle-income countries is needed as such countries are more susceptible to the possible human health risks arising from pharmaceuticals in water resources. This is as a result of the countries? dependence on surface water that might have not received any prior treatment. Lastly, there is also a need for policy development that focuses on developing water quality guidelines for pharmaceuticals in water resources. This will be the foundation for developing water quality indices that focus on the risk as well as the overall water quality status of water resources with regard to pharmaceutical pollution.

In conclusion, environmental management is a key fundamental driver of socio-economic development. The sustainable development goal (SDG) 6, set by the United Nations, for providing access to safe water and sanitation by 2030 is threatened by the emergence of new pollutants in water resources including Covid-19 drugs. To sustain water resources, pharmaceutical pollution requires close monitoring and effective management guided by research, implementation of findings, and well-developed policies. This will be best achieved through knowledge of the sources, distribution, pathways, receptors, and fate, as well as the associated risks and toxicity of the Covid-19 pharmaceuticals in water resources. This study has demonstrated the possible occurrence of azithromycin, prednisone, prednisolone, and dexamethasone Covid-19 drugs in wastewater effluent and some water resources globally. On that account, it is critical to delve more into the distribution, risks, toxicity as well as the fate of the emerging mixed pollutants of pharmaceuticals and heavy metals as such information is scares or unavailable in the current literature. The absence of pharmaceutical water quality guidelines has opened a room for pollution of water resources by pharmaceuticals. If such guidelines are in place, stringent laws and practices can be imposed on polluters such as pharmaceutical production industries, thus preventing the constant pharmaceutical pollution of water resources. This will be a step into the future that also advises on the need for much advanced methods of water treatment which will address a wide array of pollutants.

## Data Availability

No datasets were generated or analysed during the current study.
